# Repeat Length and RNA Expression Level Are Not Primary Determinants in CUG Expansion Toxicity in *Drosophila* Models

**DOI:** 10.1371/journal.pone.0001466

**Published:** 2008-01-23

**Authors:** Gwenn Le Mée, Nader Ezzeddine, Michèle Capri, Ounissa Aït-Ahmed

**Affiliations:** Institut de Génétique Humaine, Unité Propre de Recherche 1142, Centre National de la Recherche Scientifique, Montpellier, France; Lehigh University, United States of America

## Abstract

Evidence for an RNA gain-of-function toxicity has now been provided for an increasing number of human pathologies. Myotonic dystrophies (DM) belong to a class of RNA-dominant diseases that result from RNA repeat expansion toxicity. Specifically, DM of type 1 (DM1), is caused by an expansion of CUG repeats in the 3′UTR of the DMPK protein kinase mRNA, while DM of type 2 (DM2) is linked to an expansion of CCUG repeats in an intron of the *ZNF9* transcript (*ZNF9* encodes a zinc finger protein). In both pathologies the mutant RNA forms nuclear foci. The mechanisms that underlie the RNA pathogenicity seem to be rather complex and not yet completely understood. Here, we describe *Drosophila* models that might help unravelling the molecular mechanisms of DM1-associated CUG expansion toxicity. We generated transgenic flies that express inducible repeats of different type (CUG or CAG) and length (16, 240, 480 repeats) and then analyzed transgene localization, RNA expression and toxicity as assessed by induced lethality and eye neurodegeneration. The only line that expressed a toxic RNA has a (CTG)_240_ insertion. Moreover our analysis shows that its level of expression cannot account for its toxicity. In this line, (CTG)_240.4_, the expansion inserted in the first intron of CG9650, a zinc finger protein encoding gene. Interestingly, CG9650 and (CUG)_240.4_ expansion RNAs were found in the same nuclear foci. In conclusion, we suggest that the insertion context is the primary determinant for expansion toxicity in *Drosophila* models. This finding should contribute to the still open debate on the role of the expansions *per se* in *Drosophila* and in human pathogenesis of RNA-dominant diseases.

## Introduction

Myotonic dystrophy type 1 (DM1), also called Steinert myotonic dystrophy is a multi-systemic pathology originally described as a neuromuscular disorder [1]. It is an autosomal dominant disease genetically linked to a CTG trinucleotide expansion localized in the 3′UTR of *DMPK* [2]–[4]. *DMPK* encodes a serine-threonine protein kinase which phosphorylates various proteins including CUG-BP1, a CELF protein (CUG-BP/ETR3-like factor) whose dysfunction is involved in DM1 pathogenesis [5].

Essentially three models have been proposed to account for the physiopathology, the genetic inheritance and the molecular characteristics of DM1: 1) haploinsufficiency of DMPK; 2) transcription repression at *DMPK* adjacent loci; 3) toxicity of the CUG expansion containing RNA. Given the complexity of the pathology, it is possible that all these mechanisms contribute to the phenotype ([6], [7] and references therein). Arguments in favour of the RNA gain-of-function model were provided by the discovery of another myotonic dystrophy called DM2 (for type 2). DM2 is caused by a CCTG repeat expansion within the first intron of *ZN9*, a gene that encodes a zinc finger protein. This gene is not related to any of the genes that map in the vicinity of the *DMPK* locus [8], [9]. In both DM1 and DM2, the common molecular features are the RNA foci formed in the nuclei of cells from patients. The toxicity was therefore attributed to the expansion and would result from an entrapment in the RNA foci of nuclear factors, such as those involved in the splicing of specific mRNAs ([7] and references therein). Thus, the deleterious dominant effect of the CUG expanded RNA could be mediated by CUG binding proteins [10]. Indeed, defective splicing of RNA targets of the CUG binding proteins has been well documented and could explain some aspects of DM1 physiopathology [7]. CUG-BP1 was one of the first CUG binding proteins to be associated with DM1 [11], but although its level increases in DM1, it has not been detected in the nuclear foci [12], [13]. On the other hand, Muscleblind-like protein family members, such as MBNL1 in humans, bind to CUG repeats and are associated with the nuclear foci [14]. However, recent work clearly establishes that MBNL1 presence in RNA foci is not sufficient to account for the pathological effect of the expansion as MBLN1 was also found to be associated with non pathological nuclear foci [15]. The identification of another CUG expanded RNA binding factor, namely hnRNP H is more promising as in hnRNP H depeleted cells, CUG expansions do not form nuclear aggregates [16].

The finding that hnRNP H binds only to a specific sequence/structure (CUG repeat and splicing branch point distal to the repeat), demonstrates that RNA expansions acquire new properties which are dependent on the sequences in which they reside. Indeed, the basic view that nuclear aggregates formed by CUG expansions are intrinsically toxic and/or that toxicity severity is determined only by the size of the repeats and their concentration have been already challenged by various studies [6], [17]–[19]. Therefore, the mechanisms that underlie the RNA gain-of-function toxicity remain elusive and deserve to be further investigated.

To this aim, we generated fly transgenic lines to try to understand the role of repeat type, repeat length, RNA expression levels and RNA insertion context in the formation of nuclear foci and expansion toxicity. We show that CUG expansions do not have a toxic effect in most of the transgenic lines with the exception of the (CTG)_240.4_ line that develops a deleterious phenotype upon ubiquitous or neuronal induction. Furthermore, all the (CUG)_240_ and (CUG)_480_ RNAs, but not the (CUG)_16 _RNA, form nuclear foci regardless of whether they have a toxic effect or not. We also demonstrate that in the (CTG)_240.4_ line, the expansion is inserted in an endogenous gene, CG9650, that encodes a zinc finger (ZNF) protein [20], [21]. CG9650 RNA co-localizes with the CUG expansion in the nuclear foci. We thus suggest that the toxicity of this (CUG)_240.4_ expansion correlates with the fusion transcript that it forms with CG9650 RNA.

## Results

### Construct cloning and transgene stability assessment in transgenic flies

Our aim was to generate transgenic flies using the UAS/GAL4 system [22] to address the issue of the RNA gain-of-function toxicity. Specifically, we wanted to identify which repeat length (16, 240 or 480 repeats) and which type of repeat (CUG or CAG) are needed for RNA nuclear retention and toxicity. The 240 and 480 repeats used in these experiments were the interrupted repeats that have been previously used in cell models [5], [23]. They were cloned, without any coding sequence, in the *Drosophila* UASp vector. The (CTG)_16_ constructs were cloned in the SV40 3′UTR downstream the *LacZ* ORF. Five (CTG)_240_, three (CTG)_480_, one (CAG)_240,_ two (CAG)_480_ and three (CTG)_16_ transgenic lines were generated.

**Figure 1:**
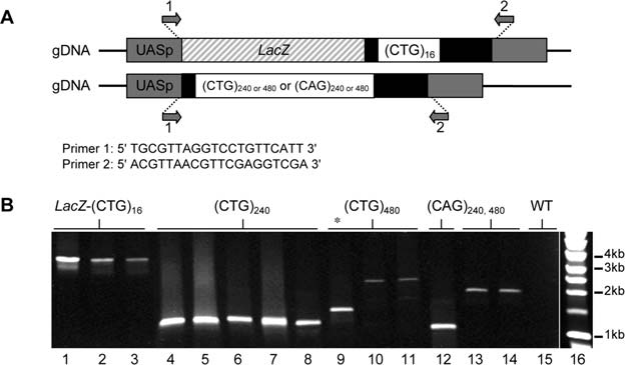
Transgenic constructs and expansion size verification. A. Organization of the (CTG)_n_ containing transgenes. Grey boxes: UASp plasmid sequences inserted in genomic DNA. White boxes: CTG repeat tracts (CTG) _n = 16, 240 or 480_ or CAG repeat tracts (CAG) _n = 240 or 480_. Black boxes: non repeated flanking sequences. For the small repeat tract (n = 16) the *LacZ* ORF is shown as a hatched box. The two primers (1 and 2) used to amplify the transgene were complementary to vector sequences and are common to all constructs. PCR amplifications required specific conditions because of the difficulty to amplify the repeat tracts especially when n = 480 (see Material and Methods). B. Gel electrophoresis analysis of PCR fragments of the transgenic flies genomic DNA amplified using primers 1 and 2. LacZ-CTG _n = 16_ (lanes 1–3), CTG _n = 240_ (lanes 4–8), CTG _n = 480_ (lanes 10–11), CAG _n = 240_ (lanes 12) or CAG _n = 480_ (lanes 13–14), wild type control DNA (WT) (lane 15). The discrepancy in the size of (CTG)_480 _and (CAG)_480 _reflects a difference in the black boxed flanking sequences, see Material and Methods. (*) partial loss of the CTG _n = 480_ tract (line 9) during the cloning process in bacteria. In Drosophila they have been stable through hundreds generations. Lane 16: 1kb DNA ladder (Promega).

First, we assessed the genomic stability of the expansions in subsequent generations, by determining their size by PCR amplification with primers complementary to sequences that were at each side of the repeats (Figure 1A). After more than 100 generations the expansion size remained unchanged indicating that the large expansions were stable in *Drosophila* (figure 1B). This is in striking contrast with their instability in bacteria, as observed during the cloning process.

### CUG repeat expansions expressed in various tissues accumulate in nuclear foci

**Figure 2:**
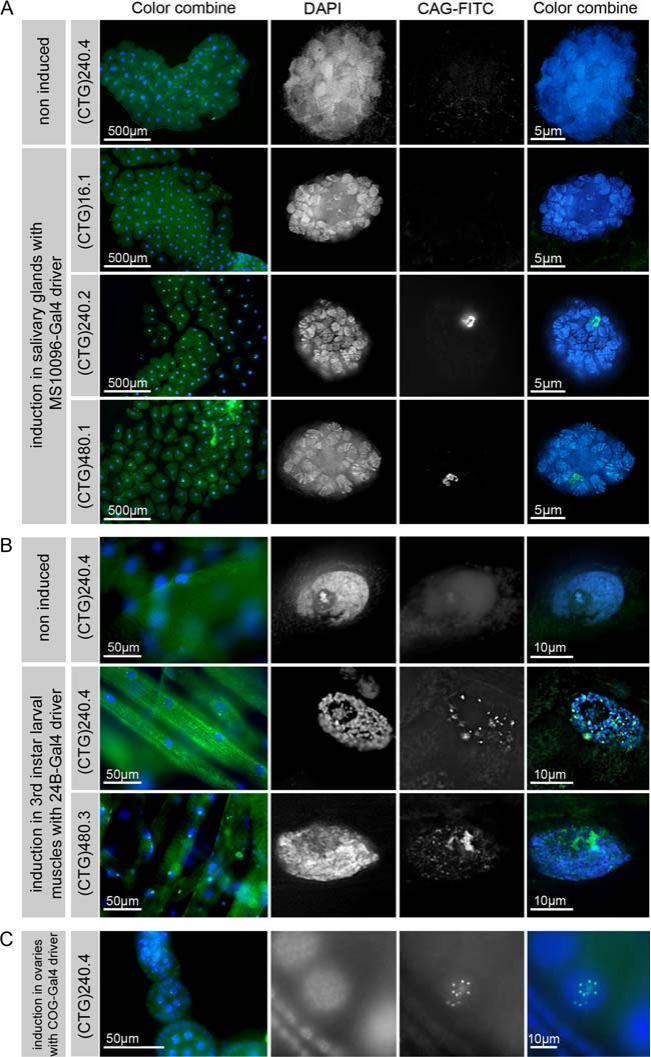
Whole mount RNA-FISH analysis using a CAG-FITC probe. UAS-(CTG)_n_ flies were crossed with *Gal4* driver flies and raised at 25°C. For transgene induction, the temperature was raised to 29°C for 12 h before dissection. A. Expression pattern of (CUG)_n_ containing RNAs induced in salivary glands by the *MS1096-Gal4* driver [24]. Upon induction, (CUG)_n_ containing RNAs formed a single nuclear focus in salivary glands of all the transgenic lines where n = 240 or 480. The nuclear signal was absent in lines with n = 16 repeats and in non induced flies. B. Expression pattern of (CUG)_n_ containing RNAs induced in muscles of third instar larvae with the *24B-Gal4* driver [22]. (CUG)_n_ RNAs formed several nuclear foci in muscles of all the transgenic lines examined where n = 240 or 480. No foci were detected when transgenes were not induced. C. Expression pattern of (CUG)_n_ containing RNAs induced in ovaries with the *COG-Gal4* driver [25]. (CUG)_n_ containing RNA formed multiple foci in all the transgenic lines where n = 240 or 480. No foci were detected in non induced flies (data not shown). DNA was stained with DAPI.

Tissue-specific induction of the repeat expansions was achieved by crossing UAS-(CTG)_n_ lines with Gal4 driver flies [22]: *MS1096-Gal4* flies for expression in the salivary glands of the third instar larvae [24], *24B-Gal4* flies for induction in the larval muscle cells [22] and *COG-Gal4* flies to induce ovary expression [25]. We tested all the (CTG)_n_ transgenic lines for (CUG)_n_ expression using RNA fluorescence hybridization (RNA-FISH) with a CAG-FITC probe (Figure 2). In salivary glands, all the induced (CUG)_240_ and (CUG)_480_ RNAs formed nuclear foci that were localized in a small area of the nucleus (Figure 2A). In contrast, in the nuclei of larval muscle cells (Figure 2B) and ovarian nurse cells (Figure 2C), we observed the presence of speckles of variable size, shape and number. The absence of hybridization in non induced lines is an indication that the signal detected by the FISH probe was not due to DNA staining. The (CUG)_16_ transgenic RNAs did not form nuclear foci (Figure 2A). Using a CTG-FITC probe, we failed to detect nuclear foci in the (CAG)_240_ and (CAG)_480_ lines (data not shown).

**Figure 3:**
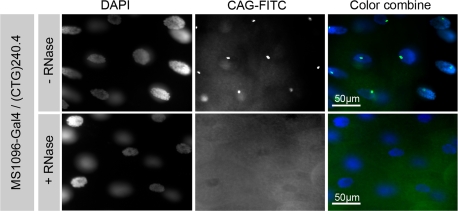
Nuclear foci formed in induced transgenic lines are RNase sensitive. The induction was performed in salivary glands as in Figure 2A. Nuclear foci formed upon driver induction were not detectable when the salivary glands were submitted to ribonuclease treatment before the FISH procedure.

To unambiguously assign to RNA the localized signal observed in the salivary glands nuclei, we treated the specimens with ribonuclease before the RNA-FISH procedure. After ribonuclease treatment, we could not detect the nuclear foci any more, whereas the DAPI signal was not affected (Figure 3).

Therefore, the nuclear foci were due specifically to the induced (CUG)_240_ and (CUG)_480_ expanded RNAs.

### A single transgenic line, (CTG)_240.4_, expresses a toxic (CUG)_240.4_ RNA

**Figure 4:**
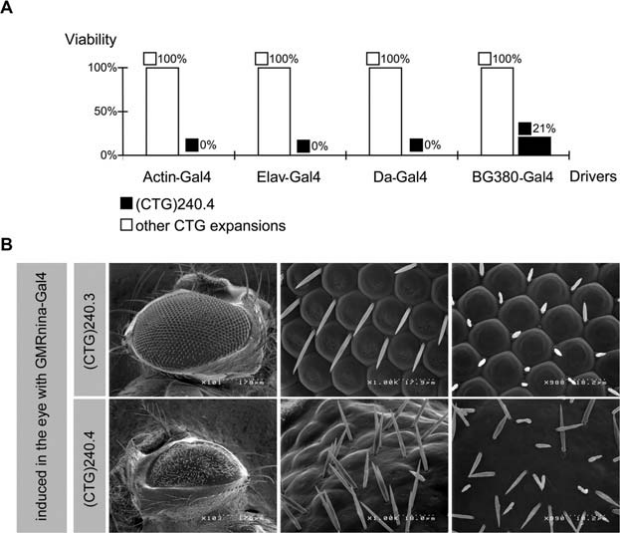
CUG expansion toxicity in *Drosophila* upon induction with various drivers. Transgene expression was achieved by raising flies that bear both UAS-(CTG)_n_ and Gal4 drivers at 25°C. A. Bar graph showing fly viability ratio in induced and non-induced conditions. Transgene expression was driven by the ubiquitous *Actin-Gal4* driver P{Act5C-GAL4}25FO1. the BL#4414 pan-neuronal *elav-Gal4* driver [26], the *daughterless-Gal4* (*da-Gal4*) driver [27] and the motoneuronal *BG380-Gal4* driver [28]. Viability was affected only in one of the (CUG)_240_ RNA expressing lines, the (CUG)_240.4_ (black boxes). No survival was observed upon ubiquitous and pan-neuronal expression and 21% viability was observed upon motoneuron induction for the (CUG)_240.4_ line. None of the other lines was affected (white boxes). B. Scanning Electron Microscopy of mutant and wild type eyes. Eye neurodegeneration upon *GMRnina-Gal4* driver induction [30] was observed only in the (CUG)_240.4_ line whereas all the other expansion lines had wild type eyes.

We used two tests to evaluate the toxicity of the RNA expansion repeats: lethality and eye neurodegeneration both induced by different *Gal4* driver lines (Figure 4). The *Actin-Gal4* constitutive driver, the *Elav-Gal4* and *Da-Gal4* pan-neural drivers [26], [27] and the *BG380-Gal4* motoneuron driver [28] were used to assess lethality. To our surprise, although all (CTG)_240_ and (CTG)_480_ lines produced nuclear foci, only the (CTG)_240.4_ had problems of viability. Indeed, upon ubiquitous and pan-neural induction the viability of this transgenic line was reduced to 0% and to 21% when the motoneuron-specific driver *BG380-Gal4* was used (Figure 4A). *BG380-Gal4* is active at larval stages, therefore, it is possible that defects resulting from the RNA toxicity hinder larval movement and feeding [28]. Similarly, only the (CTG)_240.4_ line was sensitive to induction by the *GMRnina-Gal4* driver which is specific for photoreceptor neurons [29], [30]. Eye examination using scanning electron microscopy (Figure 4B) revealed a massive disorganization of ommatidia and mechanosensory bristles, an evidence of neurodegeneration (Figure 4B).

Insertion instability/size, or a discrepancy in RNA foci formation could not provide an explanation for the limitation of RNA toxicity to a single line, (CTG)_240.4_.

### There is no correlation between expanded RNA level of expression and toxicity

**Figure 5:**
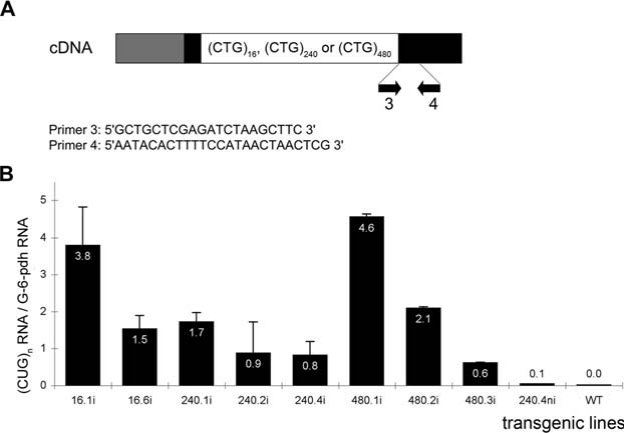
Quantitative RT-PCR amplification of (CUG)_n_ expanded RNAs. A. Organization of the (CTG)_n_ containing cDNAs. CTG repeat tracts (CTG) _n = 16, 240 or 480_ are represented as a white box. Black boxes represent 3′ and 5′UTR sequences between which they were inserted (see Material and Methods). The two PCR primers (3 and 4) used in this experiment are common to all constructs and were designed so that DNA amplification does not cross the repeat tract. B. The bar graph represents the mean ratios of CUG expanded and G-6-PDH RNA levels as determined by quantitative RT-PCR (qRT-PCR) amplification. Total RNA was extracted from heads of adult transgenic flies. *GMRnina-Gal4* driver was used for induction in the eye. Each of the transgenic lines used in this experiment is represented as a black bar. Abbreviations: i: induced; ni: non induced; wt: wild type.

To further investigate the molecular basis of (CUG)_240.4_ RNA toxicity, RNA rates were analyzed using quantitative RT-PCR (qRT-PCR) amplification. RNA extracted from adult heads of *GMRnina-Gal4* induced flies was reverse-transcribed using either oligo-dT primer or random hexamers, and cDNA was amplified using transgene-specific primers, complementary to the 3′UTR of the transcript (Figure 5A). A quantitative analysis was performed using real time PCR amplification and the expression level of each transgene was expressed as the ratio between (CUG)_n_ and G-6-PDH RNA concentrations (Figure 5B). (CUG)_n_ expanded RNA rates were highly variable even between lines that have the same repeat length. The finding that also the (CUG)_16_ lines which do not form nuclear foci, had an RNA rate variability within the same range as that of the lines with larger expansions, indicates that the RNA was quantitatively extracted from the foci. We consider that the RNA rate accounts for the level of expression.

In conclusion, the level of expression of the (CUG)_240.4_ RNA does not explain its toxicity.

### (CUG)_240.4_ forms a fusion transcript with the RNA of a zinc finger protein encoding gene

**Figure 6:**
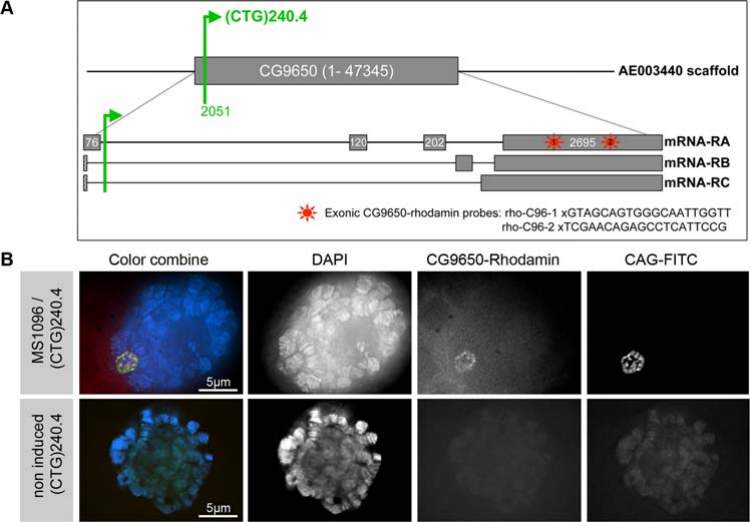
(CTG)_240.4_ expansion and CG9650 target gene expression. A. CG9650 gene organization and insertion mapping. Three CG9650 mRNAs have been reported, form A is an EST, B and C are computationally derived. Lines represent introns and exons are represented as grey boxes. The (CTG)_240.4_ transgene (green) is inserted in the first intron of CG9650 at nucleotide 2051. The position of CG9650-rhodamin probes is shown on the diagram as red stars. B. RNA-FISH on salivary glands with CAG-FITC and CG9650-rhodamin probes (rho-CG96-1 and rho-CG96-2). Nuclei were counterstained with DAPI. CG9650 RNA forms nuclear foci that co-localize with (CUG)_240.4_ RNA. *MS1096-Gal4* was used for induction. No FISH staining was detected when the transgene was not induced.

We then investigated whether the toxic effect of the (CUG)_240.4_ RNA could be accounted for by the transgene insertion context. We assessed this hypothesis by performing inverse PCR amplification on genomic DNA prepared from the different transgenic lines. All but three lines had the transgene inserted in a non transcribed region of the genome (Table 1). The (CTG)_240.4_ line was one of the three exceptions. (CTG)_240.4 _insertion lies within the first intron of the CG9650 gene that encodes a zinc finger protein [20], [21], in the same orientation as the endogenous transcription unit (Figure 6A). To gain further understanding, we investigated the expression pattern of CG9650 in the (CTG)_240.4_ line after induction of the transgene in the salivary glands using the *MS1096-Gal4* driver [24]. We performed RNA-FISH with CAG-FITC and CG9650-rhodamin probes (Figure 6A). We found that CG9650 RNA (as revealed with a probe that is complementary to exonic sequences) was concentrated in the same nuclear foci as the (CUG)_240.4 _RNA (Figure 6B). We did not observe formation of nuclear foci when CG9650 RNA was over-expressed in a context without any repeat expansion or when the RNA expansion was expressed in *trans* from an insertion that is located at a different locus (data not shown). Therefore, the (CUG)_240.4_/CG9650 foci could form only when CG9650 RNA and the CUG expansion lie in *cis* within the same transcript.

**Table 1:** Cytological and genomic mapping of the transgenic lines' insertions

Transgenic line	Chromosome	Cytogenetic map location	Gene location	Relative orientation
CTG 240.1	3	83B4	CG2922	antisense
CTG 240.2	2	53C3	intergenic	
CTG 240.3	3	86B3	intergenic	
**CTG 240.4**	**1**	**7A2**	**CG9650**	**sense**
CTG 480.1	3	67B1	intergenic	
CTG 480.2	1	5F1	intergenic	
CTG 480.3	1	2F1	CG3206	sense
CAG 240.1	1	3A5	intergenic	
CAG 480.1	3	94A2	intergenic	
CAG 480.2	2	57A7	intergenic	

Genomic mapping was performed using inverse PCR amplification as indicated in the “Material and Methods” section.

On the other hand, the (CUG)_240.1_ and (CUG)_480.3_ RNAs expressed by lines whose insertions lie also within a transcribed region (Table 1), did not have deleterious effects. No foci containing the endogenous RNAs were detected in these lines (data not shown).

The formation of a fusion RNA must be an exceptional event as a classical SV40 3′UTR was used in these constructs that brings about a functional polyadenylation signal [31]. The formation of a fusion RNA must result from a bypass of this signal. Why this occurred in the (CTG)_240.4_ line and how the fusion transcript induced toxic effects is not clear. The important issue is that the formation of a fusion RNA in the (CUG)_240.4_ line results in a situation analogous to that observed in DM1 where the expansion lies in the *DMPK* 3′UTR.

## Discussion

*Drosophila* genetics has proved to be particularly powerful in dissecting biological processes. As a result, *Drosophila* has been increasingly used to model human pathologies [32], [33] with the ultimate goal of identifying genetic modifiers and eventually finding potential new therapeutic avenues.

In the present work we wanted to generate *Drosophila* models to try to understand the molecular basis of CUG expansions toxicity.

To address this complex question we generated transgenic flies with constructs that allow expression of RNA repeats of different type and length: (CUG)_16_, (CUG)_240 and 480_, (CAG)_240 and 480_. These constructs were devoid of any of the human sequences used in various animal or cell models for myotonic dystrophy [17], [34], [35] as we wanted to concentrate our analysis exclusively on the contribution of CUG expansions to pathogenicity.

### (CUG)_240_ and (CUG)_480_ form RNA foci, most of which are not toxic in Drosophila

In this report, we confirm that CUG expansions are sufficient to generate ribonuclease sensitive nuclear foci provided they are long enough. Indeed we did not observe nuclear retention of (CUG)_16_ containing RNAs. (CAG)_240_ and (CAG)_480_ expansions did not form nuclear foci in spite of having a large number of repeats. The difference in the ability of the CAG and CUG expansions to form foci may reside in a difference in the stability of the secondary structures that are formed by these RNAs. Other Authors have described nuclear foci formation in cells expressing CAG expansions [15]. The basis for this discrepancy is unclear.

Nuclear foci have been considered as a hallmark of RNA gain-of-function toxicity. However, an increasing body of knowledge clearly suggests that the formation of nuclear foci is not sufficient [17], [19]. Indeed, formation of foci in nuclei of various fly tissues expressing (CUG)_160_ RNAs did not result in toxicity, in spite of co-localization with the Muscleblind double stranded RNA binding factor [18]. More recently, other Authors reported that induction of a (CUG)_480_ expansion in transgenic flies recapitulated at least some of the disorders associated with DM1 whereas foci formation without toxicity was observed in (CUG)_200 _expressing flies [23]. It would be tempting to conclude that the repeat length is the primary determinant of RNA expansion toxicity in *Drosophila*. However, using the same vector and repeat type [23], we did not observe toxicity upon induction of the (CUG)_480_ expansions in our transgenic lines. This was true also for all the (CUG)_240_ expressing lines but one, (CUG)_240.4_. Our results, therefore, rule out the possibility that a specific length of RNA repeats is the primary determinant in the gain-of-function toxicity in fly models.

### RNA context is the primary determinant of the CUG expansion toxicity

The (CUG)_n_ RNA expressing transgenes inserted randomly into the genome, therefore the insertion context could not be anticipated. Consequently its contribution to the RNA toxicity could be objectively assessed. Thus we were able to show that the only transgenic expansion that was toxic was expressed as a fusion transcript with an endogenous RNA, a situation that is similar to that of the RNA dominant diseases in humans. In human pathologies, expanded RNAs result from genomic amplification of endogenous repeats that lie within specific transcribed regions. In DM1, the expansion lies within *DMPK* and in DM2, it lies within *ZNF9* [2], [3], [8], [9].

One could argue that induction of toxicity in the fly transgenic lines and human pathogenesis might be supported by different mechanisms. However the *trans* acting factors that bind the CUG repeats and that are reported to be implicated in DM1 [10] have homologues in *Drosophila*, like CUG-BP1 [36], hnRNP H [37] whereas the *muscleblind* gene was first identified in *Drosophila* [38]. The similarities between fly and human CUG-binding factors point to the possibility that in DM1 pathogenesis also, the expansion *per se* might not be sufficient. The expansion context or other elements might be major determinants of the RNA toxicity and the subsequent phenotype. Not only our findings but also previously published work strengthen this hypothesis. In a cell culture model large CUG expansions, in spite of their ability to form nuclear foci, do not affect myoblast differentiation. In contrast, myoblast differentiation is affected by the same expansions when they are present in the *DMPK* 3′UTR [19], [39]. Another example comes from SCA8, an RNA *trans* dominant neurological disease in which CUG repeat expansion was implicated [40], here CUG expansion contribution to the pathology is questionable with the finding that expanded repeats are present also in healthy individuals [41]. Moreover, toxicity of SCA8 RNA when expressed in the fly is not dependent on the CUG repeat length [42].

### The molecular basis of the RNA context contribution to the phenotype is still elusive

The similarities that may be observed between the phenotypes that result from expression of CUG expansions and those that result from mis-expression of the RNA in which they reside deserve attention. Although the phenotype of the (CTG)_240.4_ line cannot be explained by a mere loss or gain of function of CG9650, both (CUG)_240.4_ RNA induction (as shown in this work), and CG9650 over-expression affect the nervous system and the eye [20], [21]. It is important that no muscular disorder was induced by the (CUG)_240.4_ expanded RNA analyzed in the present work. Similarly, in the SCA8 fly models mentioned above, no muscular disorder was induced by the CUG expansion [42].

Our hypothesis based on this work and data reported in the literature [7] is that the *trans* dominant effects of the expanded RNA may be mediated by various factors among which some must be relevant to the pathway of the RNA in which the expansion resides. In favour of this hypothesis is the observation that DM1 and DM2, the two neuromuscular disorders linked to repeat expansions in *DMPK* and *ZNF9* genes respectively, also show significant differences [3], [8]. A mouse model which presents typical symptoms of myotonic dystrophy when a normal *DMPK* 3′UTR mRNA is over-expressed [17] clearly raises issues as to the relative role of the expansion itself and of the non repeat *DMPK* 3′UTR in the pathogenicity of CUG expanded RNA in DM1 patients.

All these reports and the present one definitely challenge the view of a *trans* dominant effect of the RNA expansion, that depends only on the repeat type, size or level of expression.

Our hypothesis is, however, challenged by the finding that in DM2 no *ZNF9* RNA flanking sequences were found associated with the CCUG repeats nuclear foci [43]. Although no explanation can be provided yet, it is unlikely that the CCUG repeat expansions, differently from the CUG tracts, have an intrinsic toxicity.

These contradictory findings and the complexity of the RNA dominant diseases, suggest that the molecular mechanisms of RNA expansion toxicity are still elusive and further work is needed to properly address this question. *Drosophila* models should provide an invaluable help in solving the central question: how the mutant RNA exerts its toxic effect. Since several laboratories are working on the production of fly models of RNA dominant diseases, a number of independent insertion lines should become available to the community for a systematic investigation to be undertaken.

## Materials and Methods

### Fly stocks

UASp transgenic lines were generated as described below. For germ line transformation, experimental conditions were as described earlier [44]. Tissue specific expression of the transgenes was achieved by crossing these lines with various driver lines. *Gal4* drivers used in this work were *MS1096-Gal4* [24], *24B-Gal4* [22], *COG-Gal4* [25]. These drivers induce expression in dorsal wing disc and salivary glands, mesoderm and ovary, respectively. *Actin-Gal4* P{Act5C-GAL4}25FO1/BL#4414, *elav-Gal4*, *daughterless-Gal4* (*Da-Gal4*) and *GMRnina-Gal4* drivers were used for ubiquitous, pan-neuronal and eye expression, respectively [26], [27], [29], [30]. *BG380-Gal4* driver was used to induce expression in motoneurons [28].

### DNA constructs

#### Generation of UASp/lacZ/(CTG)_16_ transgenic constructs

The double stranded (CTG)_16_ sequence flanked by *Spe*I and *Bgl*II restriction sites was obtained by hybridizing the 5′ctagt(ctg)_16_a and 5′gatct(cag)_16_a oligonucleotides, called (CTG)_16_ and anti-(CTG)_16_. The recipient vector was prepared as following: Bluescript SK+ *Spe*I restriction site was destroyed, generating a SK/*Spe*I vector. To do so, the vector was first digested with *Spe*I, blunt ended with T4 DNA polymerase and religated. Eg5/EDEN/3′SV40 fragment from C06 plasmid [31] was then inserted in this SK/*Spe*I vector using the *EcoR*I site. The EDEN element was substituted by the (CTG)_16_ sequence described above in the SK/*Spe*I/EDEN vector, thereby becoming the SK/*Spe*I/(CTG)_16_ vector. The Eg5/(CTG)_16_/3′SV40 fragment from the SK/*Spe*I/(CTG)_16_ vector was cloned into the SK/LacZ plasmid *EcoR*I site [31]. Then a *Not*I restriction site was inserted downstream the SV40 3′UTR sequence after *Apa*I/*Xho*I digestion of the vector and integration of a linker generated by hybridization of NADXAP1 (tcgaggcggccgcgggcc) and NADXAP2 (cgcggccgcc) oligonucleotides. This new LacZ/Eg5/(CTG)_16_/3′SV40 fragment was finally cloned into UASp [25] by using the *Not*I site. The resulting UASp/lacZ/(CTG)_16_ construct was used for germ line transformation.

#### Generation of UASp/(CTG)_240 or 480_ and UASp/(CAG)_240 or 480_ transgenic constructs

Sp72(CTG)_240_ and Sp72(CTG)_480_ plasmids [5] were provided by T. Cooper (Houston, Texas). (CTG)_240 and 480_ fragments were cut out of the original plasmids by an *Xba*I/*Xho*I digestion, purified and cloned into pBluescript SK+ generating SK/(CTG)_240_ and SK/(CTG)_480_ plasmids. Flanking restriction sites were added at each side of the repeats using *Xba*I-*Spe*I-*Sal*I and *Apa*I-*Bgl*II-*Xho*I linker sequences. These modifications allow the isolation of the CTG repeats as a *Spe*I-*Bgl*II cartridge that can be used as above to replace the EDEN sequence of the SK/*Spe*I/EDEN vector. SK/(CTG)_240_/3′Eg5 and SK/(CTG)_480_/3′Eg5 plasmids were generated. The repeat fragments were then cut out as *BamH*I fragments and inserted into a *BamH*I-digested UASp vector, thus generating UASp/(CTG)_240_/3′Eg5, UASp/(CTG)_480_/3′Eg5 and UASp/(CAG)_480_/3′Eg5 plasmids depending on the insert orientation. These plasmid constructs were used for germ line transformation.

The *Xba*I-*Spe*I-*Sal*I linker was obtained by hybridizing the XSpeIS1 (ctagactagtg) and XSpeIS2 (tcgacactagt) oligonucleotides. The *Apa*I-*Bgl*II-*Xho*I linker was obtained by hybridizing the XBglIIA1 (tcgagatctgggcc) and XBglIIA2 (cagatc) oligonucleotides.

To generate UASp/(CAG)_240_/3′Eg5, a *Kpn*I fragment was inserted into the *Kpn*I site of UASp vector.

The quality of the inserts was verified twice, first by sequencing the entire fragments before their cloning into the UASp vector and then, after the last cloning step, the sequence junctions were also verified by sequencing (Genome Express). The expansions proved to be highly unstable in bacteria and all these verifications were required. The cloning procedure required to grow the bacteria at 30°C.

P element transformation was performed with the *yw* stock using standard methods [44]. Three UASp/lacZ/(CTG)_16_, five UASp/(CTG)_240_/3′Eg5, three UASp/(CTG)_480_/3′Eg5, one UASp/(CAG)_240_/3′Eg5, two UASp/(CAG)_480_/3′Eg5 independent lines were generated and were analyzed for each construct.

### Genomic DNA preparation and inverse PCR amplification

Genomic DNA (gDNA) extraction and inverse PCR amplifications were performed following the protocol provided by the “Berkeley Drosophila Genome Project Resources” (http://www.fruitfly.org). Genomic DNA was extracted from 30 flies, isopropanol precipitated and re-suspended in 150 µl TE as indicated in the BDGP procedure.

Genomic DNA (10 µl) was then digested either with *Sau*3A (Promega) or *Msp*I (Biolabs), re-ligated with T4 DNA ligase (Promega) according to the manufacturer's instructions. After ethanol precipitation, PCR amplifications were performed according to the protocol instructions both with Pwht1 and Plac1 primers for the 5′ end and with Pry4 and Pry1 primers for the 3′ end of the inserted transgene. Sequencing reactions were performed either with Sp1 for the 5′ end or with Spep1 for the 3′ end using DYEnamic ET Terminator Cycle Sequencing kit and following the manufacturer's instructions (Amersham Biosciences). Sequencing reactions were run on ABI PRISM™ 377 DNA Sequencer (Applied Biosystems).

### PCR reactions and gel electrophoresis analysis

For the LacZ (CTG)_16_ transgene, 1 µl (0.1 µg) of total DNA samples was amplified in 50 µl reactions using GoTaq® DNA polymerase according to the manufacturer's instructions (Promega). For the (CTG)_240 _or_ 480_ transgenes, 1 µl (0.1 µg) of total DNA samples was amplified in 20 µl reactions using JumpStart™ AccuTaq™ LA DNA polymerase according to the manufacturer's instructions (Sigma) with 5% DMSO and 50 mM betaine. PCR amplification reactions involved 3 cycles at 95°C (45 s), 67°C (45 s) and 72°C (1 min 30 s) followed by 30 cycles at 95°C (45 s), 62°C (45 s) and 72°C (1 min 30 s) in a MiniCycler™ (MJ Research). Amplified products (5 µl) were mixed with 1 µl of loading buffer and subjected to electrophoresis in a 1% agarose gel in 0.5× TBE with ethidium bromide and revealed by UV light. Size was determined by comparison with a 1 kb DNA ladder (Promega).

### RNA in situ hybridization (RNA-FISH) experiments

#### Tissue dissections

Salivary glands of third instar larvae were hand dissected in ice cold 1× PBT (PBS+0.1% Tween). They were fixed for 20 min in 4% paraformaldehyde in PBT, and then rinsed in PBT 5 times for 15 min. They were permeabilized for 5 min in a TE solution (10 mM Tris, 1 mM EDTA) containing 50 µg/ml proteinase K, and then rinsed twice in a PBT solution containing 2 mg/ml of glycine and twice for 10 min in PBT. A post fixation was performed for 20 min in 4% paraformaldehyde solution in PBT and then rinsed in PBT 5 times for 10 min. Salivary glands were then ready for RNA-FISH.

Muscles from third instar larvae were hand dissected. Larvae were anesthetized by placing them for 5 min on ice. Each larva was immerged in a drop of 1× PBS containing 10 mM EGTA, then opened dorsally and pinned open on a siligar covered plate with minutiens (∅ 0.15mm, Austerlitz insect pins®). The cuticles with the attached muscles were then fixed, permeabilized, after post-fixation as indicated above they were ready for the RNA-FISH procedure. The ovaries were hand dissected from 3 day-old females and treated as above.

#### RNA-FISH method

The CAG-FITC probe used was a (CAG)_6_C oligonucleotide, FITC-labelled at both ends (PROLIGO). Samples were incubated in 40% formamide, 2× SSC for 30 min and hybridized overnight at 37°C with 1 ng/µl of the CAG-FITC probe in a 200 µl final volume of a solution containing 40% formamide, 2× SSC, 0.2% BSA, 2 mM vanadyl ribonucleoside complex and 1 mg/ml yeast tRNA. Samples were then washed in 40% formamide, 2× SSC for 30 min, rinsed twice in 1× SSC in PBS for 10 min and mounted on slides with Vectashield® DAPI-containing mounting medium (H-1200, Vector). For double-labeling experiments with a CG9650 specific probe (C96), 1 ng/µl of two 5′ Rhodamin-labeled probes was added, rho-C96-1 (gtagcagtgggcaattggtt) and rho-C96-2 (tcgaacagagcctcattccg). When RNase-treated, samples were incubated for 30 min at 37°C in a solution containing 0.1 mg/ml of a DNase and Proteinase free ribonuclease A solution (Fermentas) prior to RNA-FISH.

Slides were examined under a Leica DMRA2 microscope; images were captured with a photometrics coolSNAP™ HQ camera. Images from two or three wavelengths (DAPI, FITC, Rhodamin) were assembled and colored using Metaview Software.

### RNA extraction and quantitative RT-PCR (qRT-PCR) amplifications

#### RNA extraction and quantification

Flies were raised at 29°C for induction of transgene expression by *GMRnina-Gal4* driver. 30 heads from 3 day-old flies were cut and immediately put in RNA stabilizing buffer (RNAwiz™, Ambion). Total RNA was extracted according to the manufacturer's instructions. RNA quantification was performed on 2 µl total RNA sample in a NanoDrop ND-1000 spectrophotometer (NanoDrop Technologies, Wilmington, DE).

#### Quantitative RT-PCR amplification

2 µg of each of the total RNA samples were used for first strand cDNA synthesis using Superscript™ II Reverse Transcriptase (Invitrogen™) according to the manufacturer's instructions. For real-time PCR amplification, 5 ng of cDNA template was used for (CUG)_480_ and different dilutions were used for (CUG)_240_ (10, 5, 2 ng of cDNA) in a 20 µl total reaction with 50 mM betaine, 4 mM MgCl_2_, 0.5 µM of primer 3 (gctgctcgagatctaagcttc) and primer 4 (aatacacttttccataactaactcg) and 2 µl of master mix of LightCycler® FastStart DNA Master SYBER Green I kit (Roche Diagnostics). Transgene expression levels were normalized to the level of G-6-PDH expression using G-6-PDH-C2 (cgacattcgtgacgagaagg) and G-6-PDH-R1 (gttcgaatcgttgctaacgg) primers. G-6-PDH is an endogenous housekeeping gene [45]. The qRT-PCR experiments were performed using a LightCycler instrument (Roche Diagnostics). The relative expression data were treated using the Second Derivative Maximum Method of the LightCycler® Software 3.5 (Roche Diagnostics). qRT-PCR amplification was performed at least four times for each of the (CUG)_480_ samples. For the (CUG)_240_ lines and controls, four independent experiments were carried out, with three different dilutions and two replicates for each. The mean value and standard deviation were determined using Microsoft Excel.

### Scanning Electron Microscopy (SEM)

Adult flies were frozen at −80°C, coated with Gold Palladium and examined with a Hitachi s-4000 scanning electron microscope.
